# To Cross or Not to Cross: Using MRI-Histology to Characterize Dense Collagenous Plaque in Critical Limb Ischemia

**DOI:** 10.14797/mdcvj.1186

**Published:** 2023-01-06

**Authors:** Kajol J. Shah, Bright Benfor, Christof Karmonik, Alan B. Lumsden, Trisha L. Roy

**Affiliations:** 1 School of Engineering Medicine, Texas A&M University, Houston, Texas, US; 2 Methodist DeBakey Heart & Vascular Center, Houston Methodist Hospital, Houston, Texas, US; 3 Houston Methodist Research Institute, Houston, Texas, US

**Keywords:** angioplasty, atherosclerosis, endovascular treatment/therapy, magnetic resonance angiography, MRA, magnetic resonance imaging, MRI, vascular imaging, peripheral artery disease, PAD, plaque morphology, ultrashort echo time sequence

## Abstract

Peripheral artery disease (PAD) is caused by atherosclerotic buildup in the lower extremities, leading to obstruction and inadequate perfusion to the peripheral vasculature. Impenetrable plaques initially treated with percutaneous vascular intervention (PVI) have led to worse secondary bypass outcomes and amputation in patients. In this case report, we discuss the importance of using magnetic resonance imaging (MRI) histology in PVI planning in a patient with critical limb ischemia. PVI attempts to recanalize the limb failed because of an impenetrable occlusion in the popliteal artery that was not identified on routine preoperative imaging. Subsequent bypass occluded multiple times eventually requiring an above-knee amputation. An MRI-histology protocol—using ultrashort echo time (UTE) and T2-weighted (T2W) sequences—that was performed prior to the index PVI identified the occlusion as a dense collagen plaque. Histology analysis of the amputated specimen confirmed the MRI finding. This imaging modality offers a novel approach to characterize plaque composition and morphology, thereby identifying lesions at greatest risk of PVI failure and potentially playing an important role in selecting the right candidates for an endovascular-first approach.

## Introduction

With over 236 million individuals globally affected by peripheral arterial disease (PAD), determining the appropriate interventional strategy for blood flow restoration is critical in preventing amputation and death in patients.^1^

Standard revascularization approaches include percutaneous vascular intervention (PVI) by balloon angioplasty and/or stenting as well as open bypass surgery. PVI is mostly performed as a first-line treatment due to its minimally invasive nature and low periprocedural risks.^2^ Immediate PVI failure, however, occurs in 20% of patients due to impenetrable plaques,^3^ and reintervention is required within the first year in 50% to 70% of patients with below-knee calcified plaques.^4,5,6,7^ Additionally, patients who require bypass surgery after initial PVI failure have higher amputation rates and poorer long-term patency than those who initially underwent bypass surgery.^3,8^

Hard lesions are associated with poor PVI outcomes because they prevent complete guidewire penetration and increase the likelihood of vessel wall dissections, perforations, and early elastic recoil.^9^ To prevent these adverse events, it is imperative to accurately characterize plaque morphology preoperatively to guide patient selection for PVI. Standard imaging modalities, such as duplex ultrasound and computerized tomography angiography (CTA), limit visualization of heavily calcified vessels due to imaging artifacts and variable blood flow.^10,11^ Also, x-ray angiography only provides a 2-dimensional (2D) view of the lumen, which limits its ability to accurately characterize plaque composition.^12^

Significant advances in magnetic resonance imaging (MRI) using ultrashort echo time (UTE) and T2-weighted (T2W) sequences have improved 3D visualization of plaque composition without the use of contrast and radiation. These high-resolution images can enable histologic-level characterization of plaque morphology to differentiate between hard lesions, such as calcium and dense collagen, and soft plaque components in determining the feasibility of guidewire penetration during PVI planning.^13,14^

This paper reports a case to illustrate the importance of MRI-histology in the preoperative planning of PVIs to treat critical limb ischemia.

## Case

**Figure 1:**
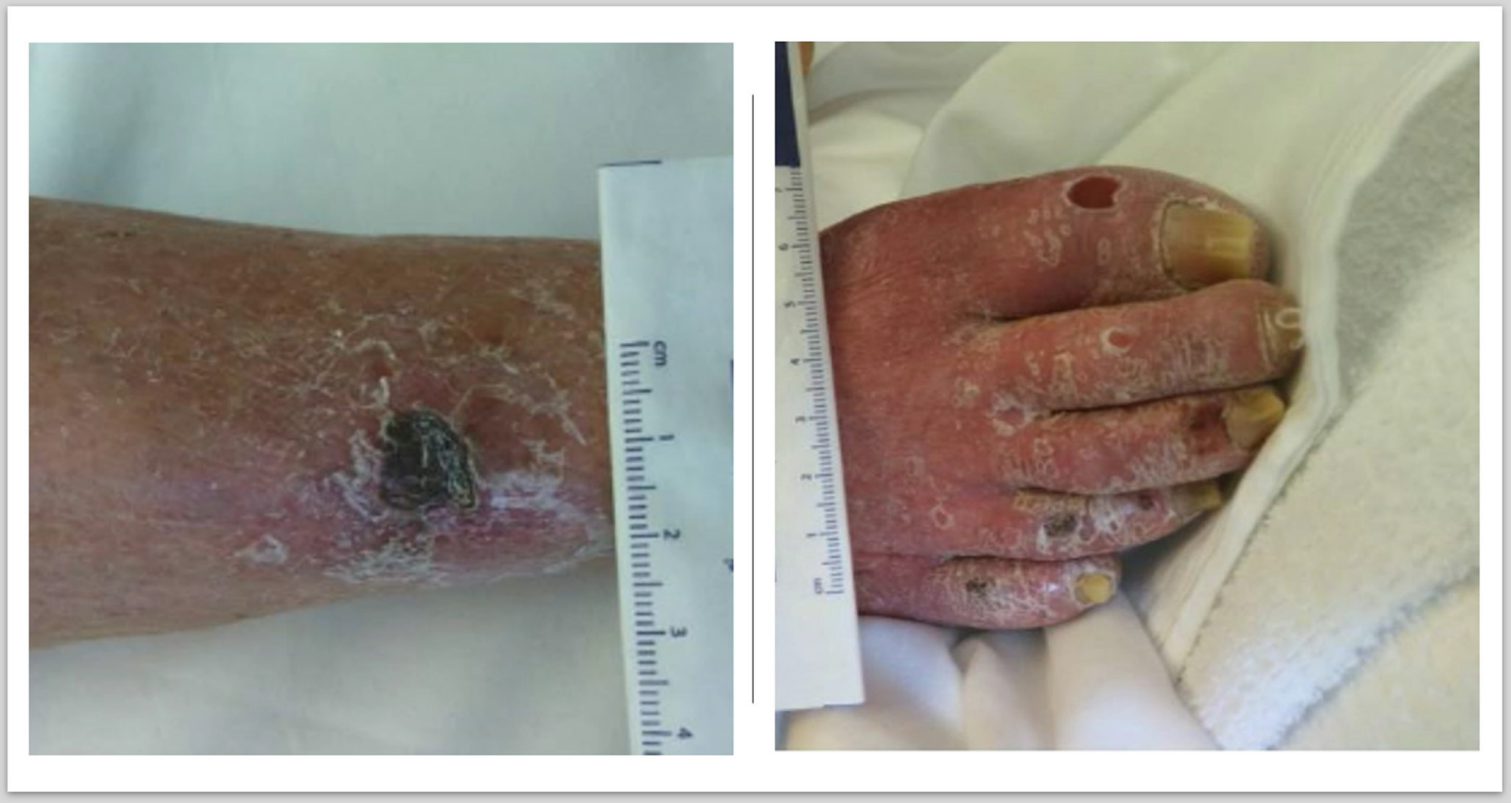
Preoperative image of right lower extremity showing necrotic ulcers in the leg and toes.

A 64-year-old female with a history of diabetes, hyperlipidemia, hypertension, coronary artery disease, and atrial fibrillation was referred to our institution for nonhealing wounds of the right foot. The patient had been nonambulatory for the past month due to worsening rest pain in her right foot. Physical examination revealed ulcers in the right leg and toes with absent pedal pulse (Figure 1).

**Figure 2:**
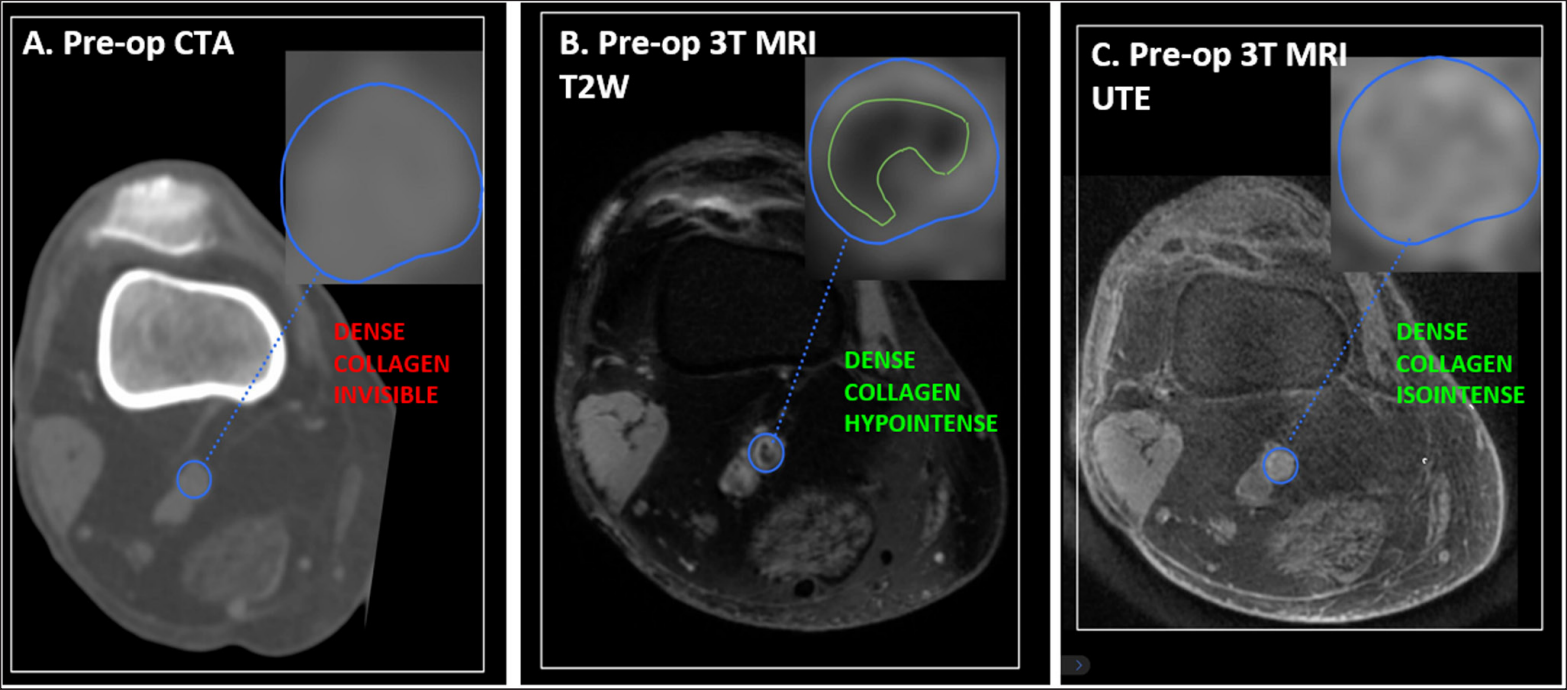
Preoperative (pre-op) imaging with computerized tomographic angiography (CTA) and magnetic resonance imaging (MRI). CTA shows an occluded popliteal artery isodense to the reference muscle tissues **(A)**. MRI shows a lesion that is hypointense in T2-weighted **(B)**and isointense in ultrashort echo time **(C)**, characteristic of dense collagen.

CTA of the lower extremities revealed a long segment of noncalcified high-grade stenosis to occlusive disease in the superficial femoral artery (SFA) (Figure 2 A), with a three-vessel runoff in the calf. The patient was also scanned with a 3T MRI as part of a research protocol that depicted a hypointense T2W signal (Figure 2 B) and an isointense UTE signal (Figure 2 C), which is characteristic of dense collagen.^13,14^ However, these MRI findings were not available to the surgeon for preoperative planning.

**Figure 3:**
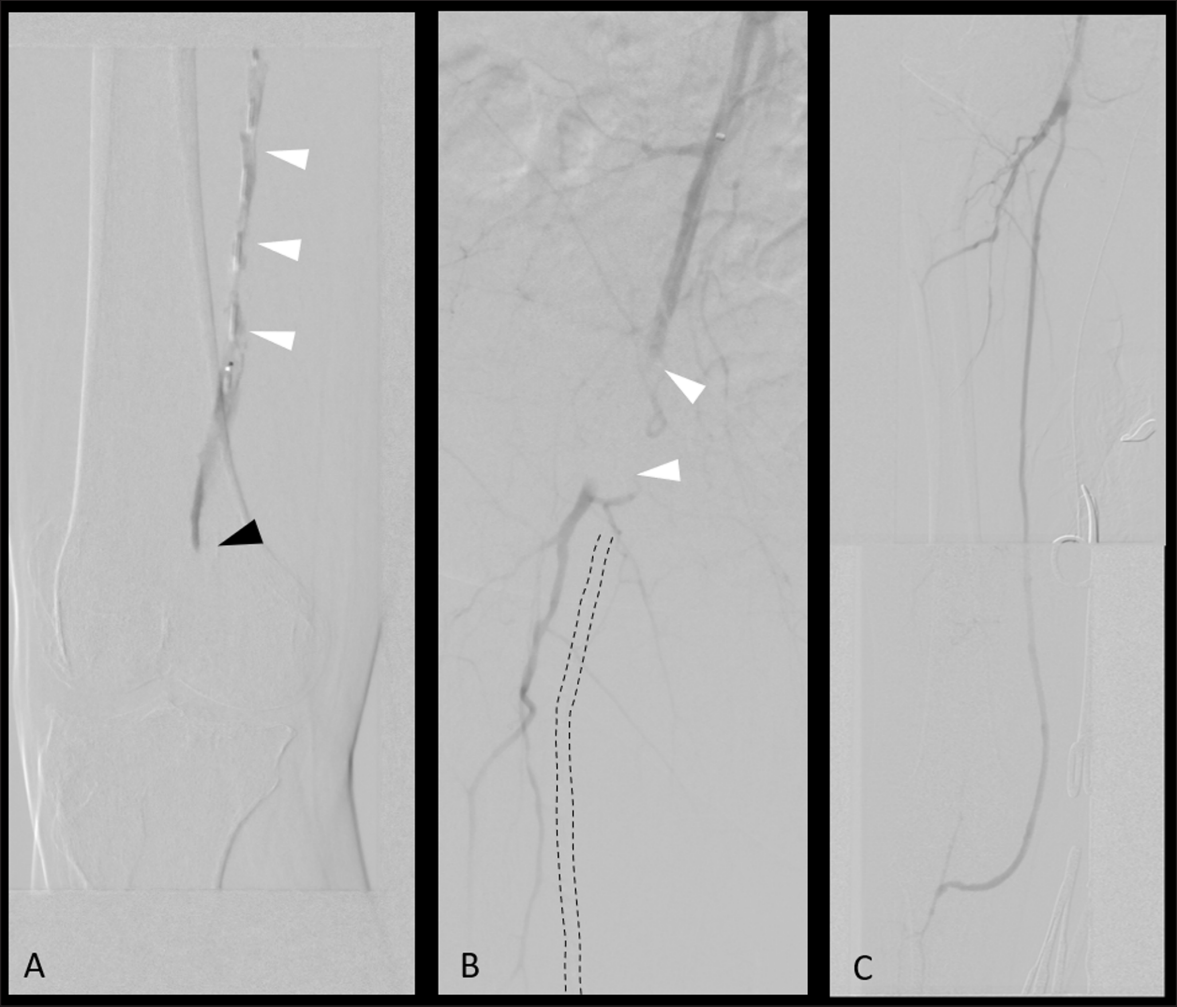
Intraoperative angiograms. **(A)** Failed attempt to recanalize occlusion (black arrow) with subintimal dissection (white arrows). **(B)** Flow-limiting dissection of the femoral artery due to multiple recanalization attempts. **(C)** Successful restoration of flow after femoropopliteal bypass.

A decision was reached to perform an arteriogram and attempt recanalization of the lesion. Thus, the patient was brought into a hybrid suite, and a crossover technique was used to access the right common femoral artery from the contralateral groin under ultrasound guidance. Several attempts to subsequently cross the SFA lesion were unsuccessful (Figure 3 A) and led to a flow-limiting dissection (Figure 3 B) that was successfully treated by balloon angioplasty. The endovascular procedure was aborted, and the patient was brought back to the operating room 4 days later for a successful right-femoral-to-below-knee popliteal artery bypass with a saphenous graft. Completion angiogram demonstrated patent bypass with no kinking and a three-vessel runoff into the foot (Figure 3 C). The patient was discharged on the sixth postoperative day with a palpable pedal pulse.

**Figure 4:**
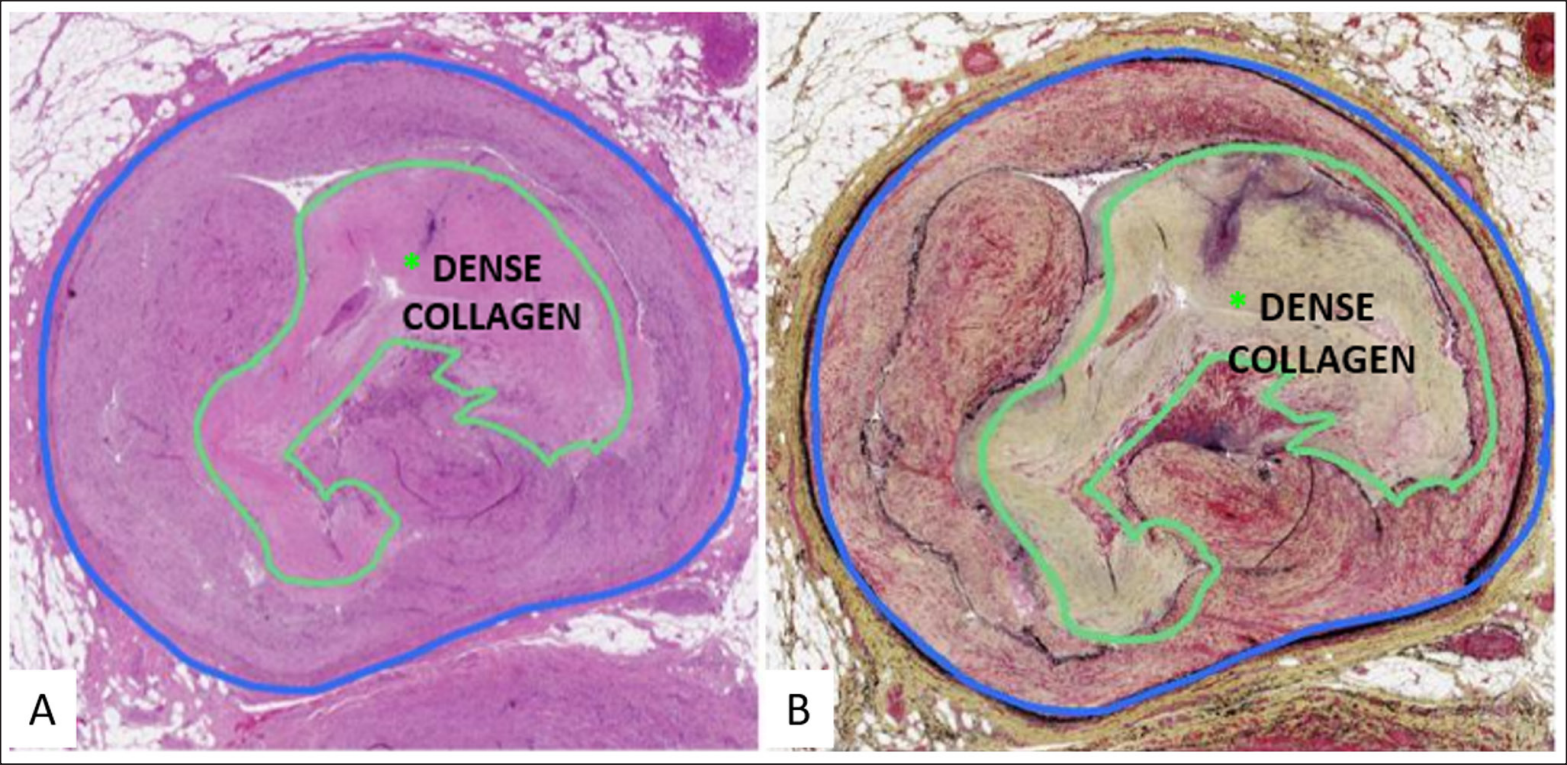
Histology of popliteal artery lesion showing dense collagen composition of atheromatous plaque.

Five months later, she presented with a 1-week history of right lower-extremity rest pain and numbness and nonhealing foot ulcers due to a bypass occlusion. This was successfully treated with a covered stent. Despite optimal wound care and medical therapy, the wounds persisted and the bypass reoccluded 3 months later, eventually leading to an above-knee amputation. The popliteal artery was harvested for histological analysis and confirmed the presence of dense collagen in the plaque (Figure 4).

## Discussion

In this case, we report on the disparities between imaging modalities in characterizing arterial plaque composition and morphology. Preoperative CTA showed a noncalcified SFA occlusion that guided decision-making to perform a PVI. Failed attempts at crossing the lesion led to further vascular insult and caused critical delays in reperfusion to the limb. Subsequent bypass failure and the need for an above-knee amputation aligns with the current evidence in literature that patients obtaining bypass after failed endovascular attempts have a worse prognosis.^3,8,15^

Advanced atheromatous plaques can be classified according to the accumulation of calcium and collagenous tissue with or without extracellular lipids over time.^16^ Preoperative MRI-histology showed an occlusion in the popliteal segment predominantly composed of dense collagen while CTA could not make this distinction. In previous studies, occlusions with dense collagen have proven to be difficult to cross with a guidewire and were associated with higher interventional failure rates.^17^ In an ex-vivo study utilizing MRI-histology, chronic total occlusions composed of dense collagen with minimal calcium took significantly longer to cross or were considered completely uncrossable. Additionally, the plaques were not discernable from soft lesions on CT or x-ray angiography.^13^

The MRI-histology protocol developed by Roy et al. uses MRI signal behavior of plaque to distinguish between soft lesions, which have slower signal decay times, and hard lesions, which have faster signal decay times.^18^ Soft plaque components are detectable on both UTE and T2W sequences. Hard plaque components such as calcium and dense collagen are detectable on UTE sequences but appear as void signals on T2W sequences.^14^ Furthermore, UTE sequences show calcium as hypointense with respect to soft tissue whereas dense collagen is isointense.^13^ In this case, MRI-histology showed an isointense signal on UTE and a void signal on T2W sequences, which is characteristic of a dense collagen occlusion. Evaluation of the MRI-histology images prior to intervention would have allowed the surgeon to identify additional hard plaque occlusions in the vessel with greater risk of crossing failure and to preemptively discuss treatment alternatives with the patient.

There is a critical need to accurately characterize plaque morphology preoperatively in patients with PAD. The combined UTE-T2W MRI-histology protocol offers a novel approach in characterizing soft/hard plaque components and enabling prediction of PVI failures in advance. Additionally, this technique offers the potential of building an anatomical scoring system that quantifies the composition and predicts the cross-ability of CTOs to allow for better-informed treatment decision-making with patients.

Common challenges with adopting MRI in practice include long acquisition times, limited availability, and greater costs as well as claustrophobia in some patients.^19,20^ MRI also requires patients to lay in supine positions for prolonged periods of time, which can be particularly difficult for critical ischemia patients because it worsens ischemic pain.^21^ Furthermore, a learning curve is associated with interpreting plaque morphology using MRI-histology since it is not traditionally used for interventional planning by vascular surgeons. Nonetheless, MRI-histology offers a safe and reliable imaging modality that provides a selective advantage in characterizing plaque morphology and reducing rates of reinterventions and major amputations.

## Conclusion

Atheromatous plaques composed of dense collagen are missed on preoperative imaging, as identified in this case. Consequent decisions to perform PVI often result in failure as dense collagen lesions are difficult to penetrate with guidewire. MRI-histology with UTE and T2W sequences can distinguish between soft plaque and hard plaque composed of calcium and dense collagen. This novel imaging technique offers a comparative edge in detecting PVI failures and selecting patients suited better for PVI versus open surgery in advance.
